# Increases in Ambulance Call Volume Are an Early Warning Sign of Major COVID-19 Surges in Children

**DOI:** 10.3390/ijerph192316152

**Published:** 2022-12-02

**Authors:** Calvin Lukas Kienbacher, Joshua Ray Tanzer, Guixing Wei, Jason M. Rhodes, Dominik Roth, Kenneth Alan Williams

**Affiliations:** 1 Division of Emergency Medical Services, Department of Emergency Medicine, The Warren Alpert Medical School, Brown University, 55 Claverick Street, Providence, RI 02903, USA; 2 Department of Emergency Medicine, Medical University of Vienna, Währinger Gürtel 18-20, 1090 Vienna, Austria; 3 Lifespan Biostatistics, Epidemiology, and Research Design (BERD) Core, 130 Plain Street, Providence, RI 02903, USA; 4 Spatial Structures in the Social Sciences (S4), Population Studies and Training Center (PSTC), Brown University, 68 Waterman Street, Providence, RI 02912, USA; 5 Center for Emergency Medical Services, Rhode Island Department of Health, 3 Capitol Hill, Providence, RI 02908, USA

**Keywords:** COVID-19, public health, emergency medical service

## Abstract

Background: Infectious diseases, including COVID-19, have a severe impact on child health globally. We investigated whether emergency medical service (EMS) calls are a bellwether for future COVID-19 caseloads. We elaborated on geographical hotspots and socioeconomic risk factors. Methods: All EMS calls for suspected infectious disease in the pediatric population (under 18 years of age) in Rhode Island between 1 March 2018 and 28 February 2022 were included in this quasi-experimental ecological study. The first of March 2020 was the beginning of the COVID-19 pandemic. We used the 2020 census tract and the most recent COVID-19 data. We investigated associations between pediatric EMS calls and positive COVID-19 tests with time series analysis and identified geographical clusters using local indicators of spatial association. Economic risk factors were examined using Poisson regression. Results: We included 980 pediatric ambulance calls. Calls during the omicron wave were significantly associated with increases in positive COVID-19 tests one week later (*p* < 0.001). Lower median household income (IRR 0.99, 95% CI [0.99, 0.99]; *p* < 0.001) and a higher child poverty rate (IRR 1.02, 95% CI [1.02, 1.02]; *p* < 0.001) were associated with increased EMS calls. Neighborhood hotspots changed over time. Conclusion: Ambulance calls might be a predictor for major surges of COVID-19 in children.

## 1. Introduction

Emergency medical service (EMS) provides essential basic access to a healthcare system. It is available for anyone at any time through a phone call. Due to this fact, the prehospital environment can be considered as the satellite of a medical system inside communities. The logical consequence might be that ambulance services see changes in community health before other aspects of the system. The latter include hospitals, and primary care providers, to which not everybody has prompt access [1]. At the same time, vulnerable populations, such as children, the destitute, and the uninsured, are at the greatest risk of suffering from the COVID-19 pandemic. The implications are multidimensional and might range from worsening economic hardship to being infected with the disease itself [2].

The United Nations International Children’s Emergency Fund (UNICEF) has found that children’s health is highly associated with their socioeconomic surroundings [2,3]. This circumstance might have been exacerbated by the continuing pandemic. Many adults lost their jobs and health insurance coverage for themselves and their dependents. Emergency department visits and admissions decreased substantially during the pandemic [1,4,5]. This might be attributed to a reluctance to walk into a hospital, triggered by the anxiety of being infected with COVID-19, or by the fear of being billed for clinic visits.

The pandemic’s impact on EMS differed globally: While some systems were overwhelmed by rapidly increasing numbers of calls, others faced the opposite [6]. The former circumstance especially increases the risk of delays in prehospital patient care, increases provider stress, and the likelihood of medical error. Ambulances arriving later in a patient’s course of illness might have the consequence that the individual is encountered in a worsened clinical condition. Related findings in the EMS providers’ assessment include the overall gestalt (i.e., providers’ impression), abnormal vital sign parameters, and an altered mental status as measured by the Glasgow Coma Scale score [7].

Because COVID-19 and its variants are highly infectious, a timely response is critical to preventing further spread. While accurate and rapid testing tools are widely available in the US, infected people may not realize that they are infected, and may be asymptomatic. Despite public health efforts, the time it takes to collect sufficient data from hospital and office providers, testing strategies to identify that an outbreak is occurring often come too late to effectively intervene. EMS requests, being closely intertwined with all communities, may act as a meaningful bellwether of upcoming outbreaks. Whether or not individuals are aware of being COVID-positive, their urgent medical needs would vary along the COVID prevalence over time, even before surveillance testing detects an outbreak. The use of EMS request data might be a helpful tool for earlier and better preparation of the healthcare system and might provide clues on how to relocate critical resources, such as personnel and isolation capacities [8].

Vector autoregressive (VAR) models can be used to elaborate the relationship between variables over time [9,10,11]. The method is well-established in economics but has become increasingly popular in the medical sciences as well [12,13]. The approach delivers more information than a single overall or one-time comparison. As effects might co-vary at different times for two variables, VAR models allow the definition of a lag, i.e., the time shift between the corresponding values of two variables. Statistical testing then allows the determination of whether one variable is helpful in explaining the timely trend of another [9,10,11].

Rhode Island is a good starting point for EMS research for many reasons: First of all, it has the smallest geographic area of all states in the United States of America, encompassing both urban and suburban areas. Secondly, its population of around one million inhabitants and its average demographic composition and socioeconomic characteristics make it comparable to many global cities [14]. Thirdly, all statewide ambulance information is comprehensively and centrally stored in the standardized National EMS Information System (NEMSIS) format on a database curated by the Rhode Island Department of Health (RIDOH) [15,16]. From there, records can be downloaded using commercial interfaces such as biospatial (Biospatial, Inc., Durham, NC, USA) and ImageTrend^TM^ (ImageTrend, Inc., Lakeville, MN, USA). The former allows the application of search filters and analysis using proprietary algorithms for syndromes, such as possible infections. The respective search accounts for categorical indications (such as disease codes), but also searches for trigger phrases within the free text narrative of the patient care report and applies proprietary analysis to accurately identify illness, injury, and other syndromes. This approach might increase the sensitivity and accuracy of the search. The definitions of the syndromes are developed in cooperation with the individual user, in our case RIDOH’s Center for EMS. In Rhode Island, EMS is available for everyone, irrespective of insurance status, and at any time via the toll-free telephone number 911.

Various approaches have been proposed to analyze geospatial data. These include methods of conventional and geospatial statistics. The latter include the Global Moran’s I and local indicators of spatial association (LISA) tests. Both examine whether data is spatially randomly distributed on a map. However, Global Moran’s I focuses on the entire area, while LISA allows investigating the spatial patterns between immediate neighborhoods regarding a certain characteristic (e.g., rates of calls between adjacent census tracts). Clusters (neighbors with similar characteristics) and outliers (areas with different properties) can be identified. This data can help to predict surges of the pandemic, which in turn allows for the proper allocation of resources. The latter include isolation and intensive care unit capacities.

We aimed to identify temporal, spatial, and demographic risk factors for flare-ups of the COVID-19 pandemic. To our knowledge, no study has yet investigated these issues using state-wide and comprehensive EMS data.

## 2. Materials and Methods

We conducted a quasi-experimental ecological study and analyzed Rhode Island’s EMS data between 1 March 2018, and 28 February 2022, retrospectively. All EMS patient records involving patients under 18 years of age were searched using biospatial screening for calls due to suspected infectious diseases. We considered 1 March 2020, the beginning of the COVID-19 pandemic. Records involving non-primary responses (e.g., interfacility transfers) and those classified as mass casualty incidents were excluded.

In the second step, the primary and secondary impressions as documented by EMS and free text narratives were screened for a possible infectious disease by a board-certified EMS physician, who is also a licensed paramedic. Records without any sign of infectious disease were excluded. Records involving patients who were likely infected with COVID-19 as deemed by ambulance crews, and those who already had a recent positive COVID-19 test according to the report were identified.

Publicly available data on COVID-19 cases, defined as individuals testing positive, was provided on a weekly basis by the Rhode Island Department of Health beginning on 1 March 2020 [17]. Due to privacy protection policies, numbers below five individuals, except zero, are blinded as “<5” [17]. To facilitate analysis, we replaced these data points with four observations. We merged the public health dataset with our EMS-derived data based on the dates of the ambulance calls. We established a multivariate time series model comprising the weekly rates of ambulance calls due to suspected infections and statewide COVID-19 cases in the population below 18 years of age.

A cross-lagged panel design was used within a time series framework [18,19]. Here we modeled the rates of pediatric COVID-19 cases and EMS calls as a function of previous rates of pediatric COVID-19 cases and EMS calls. Specifically, if rises in EMS calls precede increases in COVID-19 cases, then the number of COVID-19 cases at some week t will be positively associated with the number of EMS calls in a certain preceding week, e.g., week t − 1. The time lags and their corresponding correlation were empirically investigated within the data. Additionally, to consider the alternative possibility that the health risks of COVID-19 induce an increase in EMS calls, the model was also specified to regress EMS calls on COVID-19 cases.

While the cross-lagged effects account for the primary hypothesis and a credible alternative, other time dependencies were included in the model to control for extraneous variation that could confound results. Accounting for the possibility that the two time series may have no temporal ordering but are simply correlated, a random effect model was used to allow for a correlation between COVID-19 cases and EMS calls at week t. To account for stochastic autocorrelation, the model was specified such that previous rates of pediatric COVID-19 and EMS calls each predict current rates of pediatric COVID-19 and EMS calls respectively.

Lastly, aware that the complex transmissibility and health behavior changed over time, the number of statewide COVID-19 cases at week t was included as an independent variable for both time series. This allowed for the decomposition of the sources of variance in pediatric COVID-19 cases and EMS calls in the dynamic context of EMS response. Heterogeneous deterministic and stochastic trends were estimated for each time period. However, stochastic trends were only retained if they improved model fit as indicated by the Akaike (AIC) and Bayesian information criteria (BIC) and deviance.

In addition, we used a Poisson regression model with the statewide weekly numbers of COVID-positive children as the dependent and EMS calls as the independent variable over the entire study period. We performed a sensitivity analysis by modifying the population at risk by subtracting the individual weekly cases from the population of children.

Aware of possible confounders, i.e., lockdowns, summer camp operation guidelines in action, in-person teaching at universities, compulsory masks in schools, rollout of the vaccination program for different age groups, holidays, and the delta and omicron variant surges, we modeled relationships separately for each segment of the time period. The respective data on legal regulations were acquired from the executive orders of the Governor of Rhode Island [20]. School schedules were provided by the Rhode Island Department of Education [21]. Divisions of the study period and their dates are displayed in Table 1.

Regarding the socioeconomic risk factors, we assigned ambulance calls to census tracts based on their geolocation using ArcGIS Pro 2.9.3. Open access census tract data, including geoinformation, the economic (year 2020 median household income measured in 2020 US dollars (USD), percentage of children living in poverty), and demographic (populations under 18 years of age) surveys were downloaded from the United States Census Bureau’s homepage [22,23]. We used a Poisson regression model with the number of ambulance calls as the dependent and the economic risk factors median household income and child poverty rate as independent variables. Our model controls for the population at risk. Incident rate ratios (IRR), 95% confidence intervals (95% CI), as well as *p*-values are reported.

Global Moran’s I and LISA analyses were deployed for the entire observation period and for the corresponding sub-periods before and after the pandemic’s beginning. The underlying spatial relationship between census tracts was set to follow the Queen contiguity criterion that considers spatial units with common edges or borders as neighbors. The spatial weights matrix was row-standardized to mitigate the bias due to the differing numbers of neighbors [24]. LISA analysis was conducted with 499 permutations. We excluded census tracts with no population at risk (e.g., airport areas) and islands.

To determine the patients’ condition as stable or unstable, we dichotomized initial vital parameters into normal and abnormal, using the following cut-offs for physiological values: a body temperature ≥36.0 and ≤37.9 °C, blood sugar levels ≥70 and ≤150 mg/dL, and pulse oximetry readings of ≥95%. In order to facilitate comparability across various age groups, we defined normal heart and respiratory rates as ≥10th and ≤90th percentiles [25]. We defined altered mental status as a GCS score of fewer than 15 points. Aside from that, the EMS crew’s evaluation of the patient’s clinical gestalt, documented as “lower acuity”, “emergent” or “critical”, was taken into account. We discriminated between “emergent” and “critical” as time-sensitive and “lower acuity” as not time-sensitive. The proportions of time-sensitive patients before and since 1 March 2020, were compared using the chi-squared test.

We used ArcGIS Pro 2.9.3 (Esri corporation) for geospatial analysis and to depict the distribution of ambulance calls graphically. Stata SE 17.0 and MS Excel 16.62 (Microsoft Corporation) were used for data curation and analysis. Test results with a two-sided *p*-value of ≤0.05 were considered to be statistically significant.

The institutional review board of the Rhode Island Department of Health approved our study protocol with an exemption from full review (vote #2022-01 on 7 February 2022). The RECORD statement checklist for this manuscript is provided in Table S1.

## 3. Results

We included 980 EMS calls (460 (50%) female) in our analysis. Of these, 548 (56%) fell in the period before and 432 (44%) in the period since the beginning of the COVID-19 pandemic. The patients had a median age of 2 (IQR 1 to 6) years. Table 2 depicts the study population’s demography in detail.

### 3.1. Time-Series Analysis

**Figure 1:**
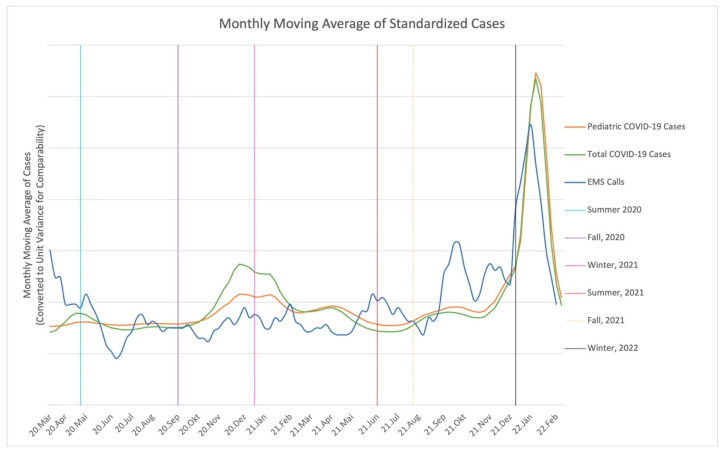
Statewide chronological trends of weekly ambulance calls due to suspected infectious diseases and positive COVID-19 test results.

**Figure 2:**
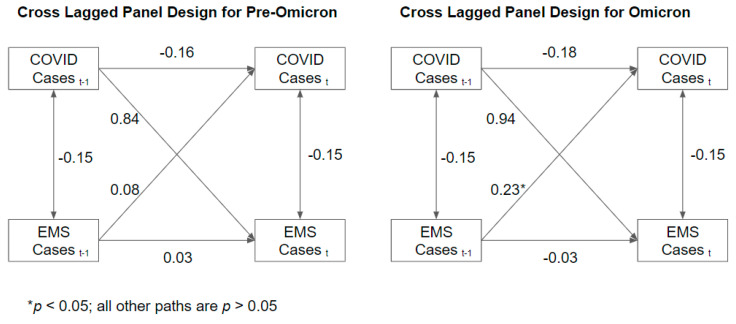
Association between ambulance calls and positive COVID-19 tests with (t − 1) and without (t) a one-week time lag.

All calls within the COVID subperiod were eligible for time-series analysis. Two heterogeneous sets of stochastic trends were retained for data analysis: from 22 August 2021 to 19 December 2021 and from 26 December 2021 to 20 February 2022, i.e., during the waves of the delta and omicron variants. A one-week time lag appeared justified by examining autocorrelation and partial autocorrelation function plots. Contrary to expectations, there was minimal association between EMS calls and pediatric COVID-19 cases during the same week (coefficient −0.15; *p* = 0.1491). Prior to the omicron variant in winter 2021/2022, none of the stochastic associations between COVID cases and EMS calls were significant (*p* > 0.05). The cross-lagged association between preceding COVID cases and future EMS calls was large and positive, but the marked swings in case prevalence caused an inconsistent association (coefficient 0.84; *p* = 0.5011). During the pediatric wave of the delta variant, we found evidence of changed health behavior, as total COVID cases were negatively associated with pediatric cases (coefficient −1.31, *p* = 0.0007). That said, during the pediatric wave of the omicron variant, increases in EMS calls tended to precede increases in COVID cases (coefficient 0.23, *p* = 0.0005). No other stochastic associations were significant (*p* > 0.05). Visually, dynamics in ambulance calls and COVID numbers appear as correlated moving average processes before demonstrating explosive increases and decreases with the apex near the end of the year 2021. The modeled deterministic trends did not indicate average increases or decreases at any point during the time series. Additionally, at no point was the correlation between the number of weekly COVID cases and EMS calls significant (*p* > 0.05). (see Figure 1 and Figure 2).

### 3.2. Poisson Regression Analysis

The analysis of the socioeconomic risk factors over the entire study period revealed that a census tract’s EMS call rate is significantly associated with its MHI (IRR 0.99; 95% CI [0.99, 0.99]; *p* < 0.001 for every 1000USD change in MHI), i.e., a 1% decrease in cases for every 1000USD increase in MHI, and its percentage of children living in poverty (IRR 1.02; 95% CI [1.02, 1.02]; *p* < 0.01 for every percent change in child poverty rate), i.e., a 2% increase in cases for every percent increase in the child poverty rate.

Independent analyses of the sub-periods before (IRR 0.99, 95% CI [0.99, 0.99]; *p* < 0.001 for every 1000USD change in MHI and IRR 1.02, 95% CI [1.01, 1.02]; *p* < 0.001 for every percent change in child poverty rate) and since (IRR 0.99, 95% CI [0.98, 0.99]; *p* < 0.001 for every 1000USD change in MHI and IRR 1.02, 95% CI [1.02, 1.02]; *p* < 0.001 for every percent change in child poverty rate) the beginning of the COVID-19 pandemic yielded similar results.

The Poisson regression model of the weekly counts of statewide pediatric COVID cases as the dependent and EMS cases as the independent variables found an association between the two variables (IRR 1.155, 95% CI [1.15, 1.16]; *p* < 0.001). These results were not altered after modifying the population at risk by subtracting the weekly cases from the overall population of minors (IRR 1.156, 95% CI [1.15, 1.16]; *p* < 0.001). Furthermore, we found a significant association between the numbers of COVID-positive adults and children (IRR 1.0001, 95% CI [1.0001, 1.0001]; *p* < 0.001).

### 3.3. Geospatial Analysis

**Figure 3:**
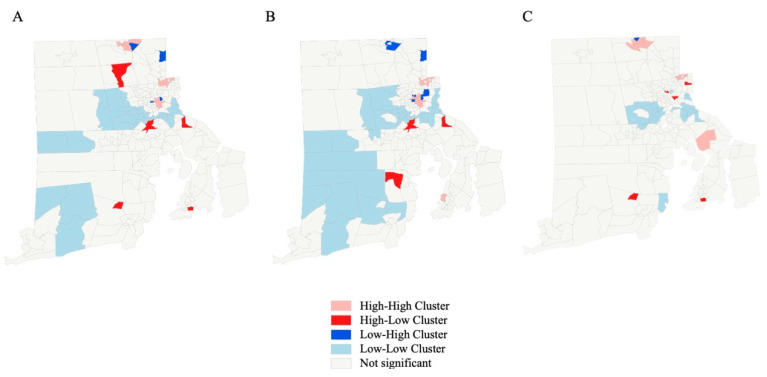
Local indicators of spatial association (LISA) for rates of ambulance calls due to suspected infectious diseases in the pediatric population on a census tract basis. (**A**), Entire observation period. (**B**), Before the beginning of the COVID-19 pandemic. (**C**), After the beginning of the COVID-19 pandemic.

Two islands were excluded from the geospatial analysis. For the remaining 244 census tracts, global Moran’s I showed clustering over the whole observation period (z-score 8.3, *p* < 0.0001), as well as before (z-score 5.2, *p* < 0.0001) and after (z-score 7.8, *p* < 0.00001) the beginning of the pandemic. The LISA analysis of ambulance call rates identified forty-nine census tracts contributing to clusters, with nine being outliers over the whole study period. Fifty cluster and fourteen outlier census tracts were detected before, vs. forty-one and six, respectively, after the beginning of the pandemic. (see Table 3 for details) The geographical redistribution indicates major shifts in neighborhood hotspots between the periods before and after 1 March 2020. (see Figure 3).

We found no differences in initial abnormal vital signs, altered mental status, or the providers’ impression between before and after the beginning of the COVID-19 pandemic. (see Table 4).

## 4. Discussion

Our data strongly indicate that dynamics in the volume of EMS calls due to various kinds of suspected infections might be a valuable predictor to foresee major surges of the COVID-19 pandemic in the pediatric population with a time lag of approximately one week in advance. Furthermore, the economic properties of neighborhoods might help to identify hotspots of increased EMS requests due to infectious diseases within immediate neighborhoods.

We are aware of the fact that our study has several limitations. First, our model is just part of the answer to how to predict flare-ups. Other information on the region’s individual characteristics (e.g., rural vs. urban, climate, etc.) must also be taken into account. Another threat to external validity might be the local COVID-19 testing policy. Disease rates detected by free, easy-access public testing might not be comparable to those derived from regions where individuals have to pay for commercial services. However, our model accounts for multiple possible confounders, including masking policies and other legal regulations.

Interestingly, our study population’s median age was two years before and after COVID-19. Toddlers are dependent on adults in daily life and do not call EMS themselves. In addition, they are often unable to use face masks properly. These circumstances imply that children of this age group are likely to either contract the disease from an adult or to infect grown-up individuals, especially their parents. Toddlers might also be an important vector for COVID-19 when kindergartens are closed, as grandparents or babysitters jump in to take care.

The temporal association between ambulance calls and COVID-19 seems to be present during major flare-ups of the pandemic and is otherwise dominated by an undirected “random walk”. These background fluctuations in both EMS calls and the number of individuals who tested positive for COVID-19 have low amplitudes and an irregular pattern. As amplitudes increase, the association seems to become stronger. With a one-week time lag, it reaches the significance level during the omicron surge at the end of the year 2021. This suggests that the value of EMS calls as a bellwether for surges to come may only be relevant during large changes in COVID-19 prevalence. We believe that it is hard to define generalizable sharp thresholds, above which trends should be considered predictive: this highly depends on individual circumstances, which change over time, such as viral mutations, other diseases (e.g., influenza), or the availability of vaccinations in a particular system. This is important because even if the specific findings of this sample may not consistently generalize, there will likely be surges of other transmissible diseases in the future. This demonstrates how changes in EMS activity can be informative during a public health crisis, even if validating thresholds for concern would be unrealistic in this sample.

Our data also show a positive association between the numbers of EMS cases and COVID-19 cases from the week prior. However, this association was not statistically significant. Following the concept that EMS is a healthcare system’s satellite within a community, it seems more plausible that increased numbers of cases are encountered in the prehospital setting before the numbers of positive test results rise than vice versa. Further, there was evidence of possible change in health behavior, because when adult COVID-19 cases were high during the delta wave, pediatric cases were low. Parents may have been trying to protect their children from infection, emphasizing the importance of using other inferential techniques to identify an outbreak than testing rates alone. Aside from that, unknown symptoms of the disease due to an infection with a mutant strand might trigger lower thresholds to call 911.

Research on the association between EMS calls and COVID-19 is still sparse. The work of Vinci et al. investigates the question of whether EMS calls can be used as a predictor for future numbers of hospitalized patients in Lazio, Italy [26]. Interestingly, they also used a time lag period for prediction of one week [26]. However, their study mainly focuses on the setting of intensive care units. Investigating the association on a more granular basis, e.g., using daily numbers, might be interesting. However, daily information on positive COVID-19 tests is not available in our setting, as these metrics are published in weekly bins only.

We hope EMS providers and public health officials can use our data to identify high-risk constellations for COVID-19 surges in time and location.

## 5. Conclusions

Our results indicate that changes in the volume of ambulance calls due to suspected infections might be used as an early warning sign for imminent flare-ups of the COVID-19 pandemic. The numeric generalizability of our results is likely to depend on a system’s accessibility of emergency medical service and testing policy. However, this has demonstrated a way to screen for worsening public health conditions that could generalize to future public health crises.

## Figures and Tables

**Table 1:** Divisions and dates within the study period, allocated to weeks of observation.

Dates (Day/Month/Year)	Calendar Period	Event
1 March 2020–5 March 2020	Spring 2020	Beginning of COVID-19 pandemic
22 March 2020–9 May 2020	Spring 2020	Lockdown
10 May 2020–13 September 2020	Summer 2020	Directive put in place for summer camps
20 September 2020–27 December 2020	Fall/Winter 2020	Virtual learning begins in schools
13 December 2020	Winter 2020/2021	Roll out of vaccination program for adults on a large scale
26 December 2020–20 February 2021	Winter 2020/2021	First pediatric cases of delta variant documented
27 December 2020	Winter 2020/2021	Roll out of vaccination program for 15- to 17-year-olds on a large scale
3 January 2021–13 June 2021	Winter 2020/Spring 2021	In-person learning begins in schools
9 May 2021	Spring 2021	Roll out of vaccination program for 10- to 14-year-olds on a large scale
20 June 2021–15 August 2021	Summer 2021	Summer begins with no safety directive for summer camp
22 August 2021–19 December 2021	Fall 2021	First pediatric cases of omicron variant documented, in-person learning begins with no mask mandate
31 October 2021	Fall 2021	Roll out of vaccination program for 5- to 9-year-olds on a large scale

**Table 2:** Characteristics of the study population, percentages rounded to integers.

	Overall (N = 980)	Before Pandemic (n = 548)	Since Pandemic (n = 432)
Female, n (%)	460 (50)	248 (45)	212 (49)
Age, years, median (IQR)	2 (1 to 6)	2 (1 to 6)	2 (1 to 7)
Childhood stages, n (%)			
Toddler (1–3 years)	446 (46)	249 (45)	197 (46)
Preschool (4–5 years)	109 (11)	63 (11)	46 (11)
Grade school (6–12 years)	148 (15)	85 (16)	63 (15)
Teenager (13–17 years)	125 (13)	60 (11)	65 (15)

**Table 3:** Results of the Local Indicators for Spatial Association (LISA) analysis for rates of ambulance calls based on census tracts, percentages rounded to integers.

	Entire Study Period (N = 244)	Before Pandemic (n = 244)	Since Pandemic (n = 244)
Cluster census tracts, n (%)	49 (20)	50 (20)	41 (17)
High-high	23 (47)	16 (32)	19 (46)
Low-low	26 (53)	34 (68)	22 (54)
Outlier census tracts, n (%)	9 (4)	14 (6)	6 (2)
High-low	5 (56)	3 (21)	5 (83)
Low-high	4 (44)	11 (79)	1 (17)

**Table 4:** Findings in primary survey, percentages rounded to integers, * Glasgow Coma Scale score below 15 points.

	Overall	Before Pandemic	Since Pandemic
Time-sensitive in clinical impression, n (%)	272 (28)	156 (28)	116 (27)
Altered mental state *, n (%)	61 (6)	31 (6)	30 (7)
At least one abnormal vital parameter, n (%)	777 (79)	457 (83)	320 (74)
Abnormal vital parameters, n (%)			
Heart rate	686 (70)	389 (71)	297 (69)
Respiratory rate	475 (48)	276 (50)	199 (46)
Pulse oximetry	55 (6)	28 (5)	27 (6)
Blood glucose	29 (3)	15 (3)	14 (3)
Body temperature	525 (54)	318 (58)	207 (48)
Handled as COVID-positive, n (%)	74 (8)	-	74 (17)
Due to EMS assumption only	33 (3)	-	33 (8)
Due to recent positive test	41 (4)	-	41 (9)

## Data Availability

Publicly available datasets were analyzed in this study. Patient-related data are not publicly available due to ethical and privacy restrictions.

## Supplementary Materials

The following supporting information can be downloaded at: https://www.mdpi.com/article/10.3390/ijerph192316152/s1, Table S1: RECORD Statement. Reference [27] is cited in the Supplementary Materials.

## References

[1] Czeisler M., Marynak K., Clarke K.E.N., Salah Z., Shakya I., Thierry J.M., Ali N., McMillan H., Wiley J.F., Weaver M.D. (2020). Delay or Avoidance of Medical Care Because of COVID-19-Related Concerns—United States, June 2020. MMWR Morb. Mortal. Wkly. Rep..

[2] United Nations International Children’s Emergency Fund Children in Monetary-Poor Households: COVID-19′s Invisible Victims. https://blogs.unicef.org/evidence-for-action/children-in-monetary-poor-households-covid-19s-invisible-victims/.

[3] United Nations International Children’s Emergency Fund Child Poverty. https://www.unicef.org/social-policy/child-poverty.

[4] Adjemian J., Hartnett K.P., Kite-Powell A., DeVies J., Azondekon R., Radhakrishnan L., van Santen K.L., Rodgers L. (2021). Update: COVID-19 Pandemic-Associated Changes in Emergency Department Visits—United States, December 2020–January 2021. MMWR Morb. Mortal. Wkly. Rep..

[5] Birkmeyer J.D., Barnato A., Birkmeyer N., Bessler R., Skinner J. (2020). The Impact of the COVID-19 Pandemic on Hospital Admissions in the United States. Health Aff..

[6] Al Amiry A., Maguire B.J. (2021). Emergency Medical Services (EMS) Calls during COVID-19: Early Lessons Learned for Systems Planning (A Narrative Review). Open Access Emerg. Med..

[7] Teasdale G., Jennett B. (1974). Assessment of coma and impaired consciousness: A practical scale. Lancet.

[8] Garcia-Castrillo L., Petrino R., Leach R., Dodt C., Behringer W., Khoury A., Sabbe M. (2020). European Society for Emergency Medicine Position Paper on Emergency Medical Systems’ Response to COVID-19. Eur. J. Emerg. Med..

[9] Granger C.W.J. (1969). Investigating Causal Relations by Econometric Models and Cross-spectral Methods. Econometrica.

[10] Hamilton J.D. (2020). Time Series Analysis.

[11] Lütkepohl H. (2005). New Introduction to Multiple Time Series Analysis.

[12] Bose E., Hravnak M., Sereika S.M. (2017). Vector Autoregressive Models and Granger Causality in Time Series Analysis in Nursing Research: Dynamic Changes Among Vital Signs Prior to Cardiorespiratory Instability Events as an Example. Nurs. Res..

[13] Yu X. (2020). Risk Interactions of Coronavirus Infection across Age Groups after the Peak of COVID-19 Epidemic. Int. J. Environ. Res. Public Health.

[14] United States Census Bureau QuickFacts Rhode Island. https://www.census.gov/quickfacts/fact/dashboard/RI/INC110219#INC110219.

[15] National Emergency Medical Services Information System (NEMSIS) What is NEMSIS. https://nemsis.org/what-is-nemsis/.

[16] NEMSIS (2020). NEMSIS Data Dictionary, NHTSA v3.4.0 Build 200910. EMS Data Standard.

[17] Rhode Island Department of Health COVID-19 Rhode Island Data. https://docs.google.com/spreadsheets/d/1c2QrNMz8pIbYEKzMJL7Uh2dtThOJa2j1sSMwiDo5Gz4/edit#gid=1199034078.

[18] Kenny D.A. (1975). Cross-lagged panel correlation: A test for spuriousness. Psychol. Bull..

[19] Cryer J.D., Chan K.S. (2008). Time Series Analysis: With Applications in R.

[20] Office of the Governor The Governor’s Archive of Executive Orders. https://governor.ri.gov/executive-order-archive.

[21] Rhode Island Department of Education School Calendar. https://www.ride.ri.gov/StudentsFamilies/RIPublicSchools/SchoolCalendar.aspx.

[22] United States Census Bureau TIGER/Line Shapefiles. https://www.census.gov/geographies/mapping-files/2020/geo/tiger-line-file.html.

[23] United States Census Bureau Explore Census Data. Advanced Search. https://data.census.gov/cedsci/advanced.

[24] ESRI ArcGIS Pro. Modeling spatial relationships. https://pro.arcgis.com/en/pro-app/latest/tool-reference/spatial-statistics/modeling-spatial-relationships.htm#GUID-DB9C20A7-51DB-4704-A0D7-1D4EA22C23A7.

[25] Fleming S., Thompson M., Stevens R., Heneghan C., Plüddemann A., Maconochie I., Tarassenko L., Mant D. (2011). Normal ranges of heart rate and respiratory rate in children from birth to 18 years of age: A systematic review of observational studies. Lancet.

[26] Vinci A., Pasquarella A., Corradi M.P., Chatzichristou P., D’Agostino G., Iannazzo S., Trani N., Parafati M.A., Palombi L., Ientile D.A. (2022). Emergency Medical Services Calls Analysis for Trend Prediction during Epidemic Outbreaks: Interrupted Time Series Analysis on 2020–2021 COVID-19 Epidemic in Lazio, Italy. Int. J. Environ. Res. Public Health.

[27] Benchimol E.I., Smeeth L., Guttmann A., Harron K., Moher D., Petersen I., Sørensen H.T., von Elm E., Langan S.M., The RECORD Working Committee (2015). The REporting of studies Conducted using Observational Routinely-collected health Data (RECORD) Statement. PLoS Med..

